# Causal relationship between thyroid dysfunction and ovarian cancer: a two-sample Mendelian randomization study

**DOI:** 10.1186/s12885-024-12385-5

**Published:** 2024-05-23

**Authors:** Tingting Wang, Xiaoqin Wang, Jun Wu, Xin Li

**Affiliations:** Department of Gynecology, Women and Children’s Hospital Affiliated to Ningbo University, Ningbo, Zhejiang China

**Keywords:** Mendelian randomization, Thyroid function, Hyperthyroidism, Ovarian cancer, Causality

## Abstract

**Purpose:**

Observational studies and clinical validation have suggested a link between thyroid dysfunction and an elevated ovarian cancer (OC) risk. However, whether this association indicates a cause-and-effect relationship remains uncertain. We aimed to investigate the plausible causal impact of thyroid dysfunction on OC through a Mendelian randomization (MR) study.

**Methods:**

Genome-wide association study (GWAS) data for thyrotropin (TSH), free thyroxine (FT4), hypothyroidism, and hyperthyroidism were obtained as exposures and those for OC (*N* = 199,741) were selected as outcomes. Inverse variance-weighted method was used as the main estimation method. A series of sensitivity analyses, including Cochran’s Q test, MR-Egger intercept analysis, forest plot scatter plot, and leave-one-out test, was conducted to assess the robustness of the estimates.

**Results:**

Genetic prediction of hyperthyroidism was associated with a potential increase in OC risk (odds ratio = 1.094, 95% confidence interval: 1.029–1.164, *p* = 0.004). However, no evidence of causal effects of hypothyroidism, TSH, and FT4 on OC or reverse causality was detected. Sensitivity analyses demonstrated consistent and reliable results, with no significant estimates of heterogeneity or pleiotropy.

**Conclusions:**

This study employed MR to establish a correlation between hyperthyroidism and OC risk. By genetically predicting OC risk in patients with hyperthyroidism, our research suggests new insights for early prevention and intervention of OC.

**Supplementary Information:**

The online version contains supplementary material available at 10.1186/s12885-024-12385-5.

## Introduction

Ovarian cancer (OC) ranks among the three major gynecological malignancies in women and stands as the second leading cause of death worldwide [1, 2]. According to the National Cancer Institute’s most recent SEER Recorded Epidemiological Survey, the annual incidence of OC is 11.6 cases per 100,000 women [3]. Unfortunately, detecting OC in its early stages is often challenging, with 60% of cases being diagnosed at an advanced stage. In 2020 alone, the United States reported over 21,000 new cases and 13,000 deaths [4, 5]. With a 5-year overall survival rate of < 30%, OC holds the highest mortality rate among female reproductive system tumors [6]. Despite major advancements in clinical surgical technology, such as laparoscopy and the Da Vinci robotic system, survival rates for patients with OC have not improved. Consequently, OC has become a global health issue for women.

The pathogenesis of OC remains poorly understood. Reported risk factors include genetic factors, endocrine factors, fertility factors, endometriosis, and environmental factors [7–9].The thyroid, as the principal endocrine gland of the human body, synthesizes and secretes thyroid hormones crucial for regulating various fundamental functions such as human growth, differentiation, development, and metabolism. These hormones are essential to the human body. Recent studies have indicated that thyroid hormones possess cancer-promoting effects across various cancers by promoting cell proliferation and differentiation [10, 11]. Consequently, thyroid dysfunction has been categorized as a potential risk factor for cancer development and prevention. Currently, evidence-based data regarding the impact of thyroid hormone and its regulatory hormones on function of the ovary, a crucial female reproductive endocrine organ, remain unclear. Existing evidence on the association between thyroid function and OC primarily stems from observational studies. A nested case-control study revealed no association between hyperthyroidism and ovarian malignancy, whereas hypothyroidism was associated with the occurrence of ovarian malignancy [12]. Another study utilizing high-sensitivity chemiluminescence reported that hypothyroidism did not influence OC progression and prognosis [13]. The results of these different approaches have been contradictory, making it difficult to establish a causal relationship between thyroid dysfunction and OC.

The Mendelian randomization (MR) method, employed in this study, addresses this challenge. MR serves as an innovative epidemiological approach utilizing genetics to examine causality for exposure–outcome associations [14]. The advantage of MR over observational studies lies in its ability to overcome reverse causality and confounding factors [15, 16]. In this study, we used Mendelian stochastic analysis to explore the causal relationships among hyperthyroidism, hypothyroidism, TSH, FT4, and OC risk, aiming to further investigate the etiology of OC and provide novel insights into its clinical prevention and treatment.

## Methods

### Study design

In this study, we employed a two-sample MR to evaluate the causal link between thyroid dysfunction and OC risk. We used summary data from genome-wide association studies (GWASs) of European ancestry cohorts.GWAS summary statistics were obtained to extract prominent single nucleotide polymorphisms (SNPs)serving as genetic instrumental variables(IVs) for thyroid dysfunction and OC.we designated the TSH, FT4, hyperthyroidism, and hypothyroidism as the exposure and OC as the outcomes to ascertain their potential roles in either inhibiting or fostering the onset of OC.Adherence to three fundamental assumptions is crucial in ensuring the reliability of the results in every MR analysis:1)genetic variation is associated with the exposure of interest; 2) genetic variation is independent of confounding factors; and 3) genetic variation affects outcome only through the exposure of interest [17]. As shown in the Supplementary Fig. 1. STROBE-MR guidelines were used to guide the design of this study [18] (Supplementary Table SS1).

### Data source

Data were primarily obtained from the IEU OpenGWAS database and The ThyroidOmics Consortium database. Our exposures of interest were TSH, FT4, hyperthyroidism (decreased TSH), and hypothyroidism (increased TSH). We obtained the GWAS data for hypothyroidism (GWAS ID: ebi-a-GCST90018862) and hyperthyroidism (GWAS ID: ebi-a-GCST90038636) from the IEU database (https://gwas.mrcieu.ac.uk/). The hypothyroidism group consisted of 30,155 cases and 379,986 controls, totaling 410,141 samples. Similarly, The hyperthyroidism group included 3,731 cases and 480,867 controls, totaling 484,598 samples. We sourced the GWAS data for TSH and FT4 from The ThyroidOmics Consortium database(https://transfer.sysepi.medizin.uni-greifswald.de/thyroidomics/datasets/), which included a total of 72,167 samples [19].

As for the outcome, we selected OC (GWAS ID: ieu-b-4963) and obtained its GWAS data from the IEU database (UK Biobank (https://gwas.mrcieu.ac.uk/). The OC group comprised 1,218 cases and 198,523 controls, totaling 199,741 samples. All the summary data used in this study are publicly available and we have obtained ethical permissions from the respective institutional review boards. (Table 1)

**Table 1:** Source and related information of instrumental variables

Variable	Sample size	Total SNPs	Selected SNPs	Population	Consortium
Hyperthyroidism	484,598	9,587,836	17	European	IEU open database
Hypothyroidism	410,141	24,138,872	66	European	IEU open database
TSH	72,167	7,958,096	26	European	hyroidOmics Consortium database
FT4	72,167	7,963,150	11	European	hyroidOmics Consortium database
Ovarian cancer	199,741	9,822,229		European	UK Biobank

FT4;free thyroxine; TSH, thyrotropin; IEU, IEU open database

### Instrumental variables

In this study, we rigorously selected effective instrumental variables (IVs) following the three assumptions: First, we ensured that each IV exhibited a strong correlation with the exposure (*p* < 5 × 10^− 8^). To address linkage disequilibrium between each SNP, we set a physical distance threshold of 10 Mb and an LD *r*^2^ to < 0.001 [20, 21]. we utilized the PhenoScanner v2 database (available at: http://www.phenoscanner.medschl.cam.ac.uk/) to identify and exclude IVs that might influence the potential level of pleiotropy in SNP-associated phenotypes [22]. we also harmonized the SNPs for exposure and outcome, removing palindromic and incompatible alleles [23]. Finally, we calculated the F-statistic for each SNP, where weak IVs (F < 10) were excluded. To guarantee the robustness of the association between the IVs and exposure factors [24]. The F-statistic was calculated using the formula: F = (*N* − 2) × R^2^ / (1 − R^2^); R^2^ = β^2^ × (1 − EAF) × 2EAF, where R^2^ is the degree of variation explained by each SNP, EAF is the gene frequency of the mutation, β is the beta coefficient associated with the exposure factor, and N is the total sample size [25].

### MR analysis and sensitivity analyses

The inverse variance-weighted (IVW) method was used as the primary analysis in this study to initially assess the potential causal thyroid effect of dysfunction on OC [26]. Assuming the validity of all selected IVs, IVW demonstrated the most reliable result and highest statistical power [27]. Moreover, complementary approaches such as weighted mode, weighted median method, the simple median method, and MR-Egger for multiple genetic variants were employed to assess the causal effect [28–30]. In addition, we performed a variety of sensitivity analyses for significant or nominally significant results.We used Cochran’s Q test and MR multivariate residual sums and outliers (MR-PRESSO) test to calculate the potential heterogeneity, where *p* < 0.05 indicated the presence of heterogeneity [31]. The intercept value of MR-Egger regression was used to assess the strength of horizontal pleiotropy, with a significance level of *p* > 0.05 suggesting no horizontal pleiotropy [29]. Additionally, the robustness of the results was assessed using the leave-one-out test to determine if the inclusion of a single SNP influenced the robustness of the findings [32]. As a complement, scatter plots were used to observe the consistent effects estimated by the five methods.The results are presented as odds ratios (OR) with 95% confidence intervals, and statistical significance was defined as *P* < 0.05. Statistical analyses were performed using two-sample MR analyses of thyroid dysfunction and OC with the two-Sample MR packages in R (version 4.3.1).

## Result

### Genetic variants selection

In this study, SNPs with linkage imbalance and palindromic structure were excluded. After conducting a series of quality evaluations, we selected a total of 17, 66, 26, and 11 SNPs as effective instrumental variables (IVs) for hyperthyroidism, hypothyroidism, TSH, and FT4, respectively. Additional information on these SNPs as IVs can be found in the Supplementary Material (Supplementary Tables S1–S4). It is worth noting that all the SNPs used as IVs had an F-statistic > 10 (Supplementary Tables S1–S4), indicating their effective performance.

### Causal effect of thyroid function on ovarian cancer

We employed several methods including simple mode, MR-Egger, weighted mode, weighted median, and IVW to assess the presence of a causal relationship between thyroid function and OC risk. Our IVW analysis revealed a significant correlation between hyperthyroidism levels and an elevated risk of OC (OR = 1.094, 95% CI: 1.029–1.164, *p* = 0.004). Consistent results were also observed with MR-Egger (OR = 1.174, 95% CI: 1.054–1.308, *p* = 0.011), weighted median (OR = 1.148, 95% CI: 1.057–1.245, *p* = 0.001), and weighted mode (OR = 1.133, 95% CI: 1.044–1.229, *p* = 0.011) methods. Conversely, our MR analysis did not find a statistically significant causal relationship between hypothyroidism, TSH, FT4, and OC risk. (Table 2; Supplementary Fig. 2)

**Table 2:** MR estimates from different methods of assessing the causal effect of thyroid dysfunction on OC

Exposure	MR methods	nSNP	Beta	SE	OR(95% Cl)	*P*-value
Hyperthyroidism	MR Egger	17	0.160	0.055	1.174(1.054,1.308)	0.011
	Weighted median	17	0.138	0.041	1.148(1.057,1.245)	0.001
	IVW	17	0.090	0.032	1.094(1.029,1.164)	0.004
	Simple mode	17	0.120	0.068	1.128(0.987,1.289)	0.108
	Weighted mode	17	0.125	0.042	1.133(1.044,1.229)	0.011
Hypothyroidism	MR Egger	66	9.599e-04	0.001	1.001(0.999,1.002)	0.208
	Weighted median	66	6.291e-04	0.001	1.001(0.999,1.002)	0.234
	IVW	66	6.201e-05	0.000	1.000(0.999,1.002)	0.857
	Simple mode	66	4.859e-04	0.001	1.000(0.998,1.003)	0.669
	Weighted mode	66	7.929e-04	0.001	1.0001(0.999,1.002)	0.274
TSH	MR Egger	26	-0.003	0.003	0.997(0.991,1.002)	0.241
	Weighted median	26	-0.001	0.001	0.999(0.997,1.002)	0.700
	IVW	26	<0.001	0.001	1.000(0.999,1.002)	0.747
	Simple mode	26	-0.001	0.003	0.999(0.994,1.004)	0.697
	Weighted mode	26	-0.002	0.002	0.998(0.993,1.003)	0.489
FT4	MR Egger	11	2.194e-03	0.002	1.002(0.997,1.007)	0.393
	Weighted median	11	-6.022e-06	0.002	0.999(0.997,1.003)	0.997
	IVW	11	-1.967e-04	0.001	0.999(0.997,1.002)	0.873
	Simple mode	11	9.234e-04	0.003	1.001(0.995,1.007)	0.781
	Weighted mode	11	2.246e-04	0.002	1.000(0.997,1.004)	0.903

SNP, single nucleotide polymorphism; TSH, thyrotropin; FT4,free thyroxine; MR, Mendelian randomization; IVW, inverse variance weighting.

### Sensitivity analysis, heterogeneity, and pleiotropy

No heterogeneity was noted among SNPs when hyperthyroidism was used as the exposure (Cochran’s Q value = 12.773, *P* = 0.689). MR-PRESSO also did not find outliers with excessive heterogeneity.Moreover, heterogeneity was not found in hypothyroidism (*P* = 0.279), TSH (*P* = 0.561), and FT4 (*P* = 0.473). The MR-Egger regression analysis indicated that there was no horizontal pleiotropy in any of the exposures, as shown in (Table 3).These findings suggest that IVs do not significantly influence outcomes through mechanisms other than the exposure. The stability of the results was further confirmed by the leave-one-out test, as demonstrated in (Supplementary Fig. 3). Scatter plots suggested consistent direction of multiple methods and the funnel plots were also symmetrical (Supplementary Fig. 2; 4), while Supplementary Fig. 5 presents the forest plots in the MR analysis. Therefore, we consider the results obtained from the IVW method to be reliable.

**Table 3:** Sensitivity analysis of correlation between exposure (thyroid dysfunction) and ovarian cancer (OC)

Exposure	Outcome	Pleiotropy		Heterogeneity		MR-PRESSO
		Horizontal pleiotropy (Egger intercept)	Horizontal pleiotropy(*P*-value)	Heterogeneity *(Q*)	Heterogeneity(*P*-value)	
Hyperthyroidism	OC	-0.001	0.141	12.773	0.689	0.722
Hypothyroidism	OC	-0.001	0.186	71.211	0.279	0.787
TSH	OC	0.001	0.170	23.281	0.561	0.741
FT4	OC	-0.001	0.286	9.636	0.473	0.714

TSH, thyrotropin; FT4,free thyroxine; OC, Ovarian cancer.

## Discussion

In this study, we used a two-sample MR analysis to investigate, for the first time, the association between thyroid function and OC. The results indicated that hyperthyroidism increases OC risk, whereas no association was observed between hypothyroidism, TSH, and FT4 and OC risk.

The significant increase in OC risk with hyperthyroidism, as found in our study, aligns with previously published findings. For instance, a clinical study demonstrated a significant association between hyperthyroidism and OC risk [33], contrasting with findings showing no association with hypothyroidism [34]. Similarly, a cohort study of Asians (including 115,746 participants) revealed a link between hypothyroidism and increased cancer incidence and mortality [35]. However, conflicting results have been reported in other studies. A nested case-control study found no correlation between hyperthyroidism and ovarian malignancy, whereas hypothyroidism was correlated with the occurrence of ovarian malignancy [12]. Another study using high-sensitivity chemiluminescence found that hypothyroidism does not affect OC progression and prognosis [13]. Previous observational studies have certain methodological limitations, including sample size, study population, and confounding factors that are difficult to control. These limitations can yield heterogeneous results. In this study, the MR method was employed to verify the correlation between thyroid dysfunction and OC from the perspective of IVs, yielding more universal and robust results.

Abnormal thyroid hormone levels exert significant effects on female reproductive endocrinology, strictly controlled by the hypothalamic-pituitary-thyroid axis [36]. Abnormal thyroid function can cause various diseases, including atrial fibrillation and abnormal lipid metabolism [37, 38]. Observational studies have found associations among thyroid hormone levels, related diseases, and cancer risk, including colorectal, prostate, and lung cancers [ [35, 39–42]]. Recent studies have specifically linked hyperthyroidism to OC. However, the exact mechanism linking thyroid dysfunction and OC has not been fully elucidated. Two main mechanisms of action of thyroid hormones have been identified through cell experiments. The first involves non-genomic action, where thyroid hormones (T3 and T4) interact with integrin αvβ3 to activate protein kinase/extracellular signal-regulated kinase pathways [43]. Activation of these signaling pathways can promote cell proliferation, thereby promoting cancer progression and inhibiting apoptosis and metastasis [ [44–46]]. The second mechanism involves the binding interaction of thyroid hormone with nuclear thyroid hormone receptor proteins, inducing transcription and activating or inhibiting various downstream effects of target genes [47]. Thus, abnormal thyroid function may activate associated cancer signaling pathways, potentially increasing OC risk. Our study observed a positive causal association between hyperthyroidism and OC but found no association between the other three exposure factors (TSH, hypothyroidism, and FT4) and OC. However, previous observational studies have reported increased OC risk or associated prognosis with hypothyroidism [48]. This inconsistency may stem from methodological differences between studies, particularly potential confounding factors present in observational studies.

Our study’s strengths include using MR studies for causal reasoning and selecting genetic variation as IVs, independent of each other, helping to avoid the interference of other reverse causation and confounding factors, thereby reducing bias. Moreover, we employed various stable methods, such as IVW and MR-Egger, to obtain reliable results. However, our study has some limitations. First, all participants were of European descent, potentially limiting the generalization of findings to other races and ethnicities and introducing bias. Second, due to our strict threshold, certain genetic defects in thyroid function were excluded at the IV selection stage, potentially missing some results. Third, due to research limitations, we cannot distinguish between different types of OC.

## Conclusions

In summary, based on analysis of data from the Thyroid Consortium database and the UK Biobank, this study suggests a causal relationship between hyperthyroidism and OC, underscoring the importance of thyroid hormones in the prevention and treatment of the female reproductive system. However, the mechanism by which the hypothalamic-pituitary-thyroid axis promotes OC development remains incompletely understood and requires further study.

### Electronic supplementary material

Below is the link to the electronic supplementary material.

Supplementary Material 1

Supplementary Material 2

Supplementary Material 3

## Data Availability

Data is provided within the manuscript or supplementary information files.

## Abbreviations

OC: Ovarian cancer
MR-PRESSO: Mendelian randomization pleiotropy residual sum and outlier
MR: Mendelian randomization
IVW: inverse variance weighted
WM: weighted modelling method
SM: simple mode
WME: weighted median estimation model
IVs: instrumental variables
TSH: thyrotropin
FT4: free thyroxine
GWASs: genome-wide association studies
