# Supplementary material for: Causal relationship between thyroid dysfunction and ovarian cancer: a two-sample Mendelian randomization study
Source: BMC Cancer. 2024 May 23;24:629. doi: 10.1186/s12885-024-12385-5 (PMC11112802; doi:10.1186/s12885-024-12385-5)
Supplement: Supplementary file 2 — Supplementary Material 2 [file 12885_2024_12385_MOESM2_ESM.docx]

Supplementary Figure

# Article title:Causal relationship between thyroid dysfunction and ovarian cancer: A two-sample Mendelian randomization study

# Journal name: JOURNAL OF CANCER RESEARCH AND CLINICAL ONCOLOGY

Author names: Tingting Wang^1^, Xiaoqin Wang^2*^ ,Jun Wu^3^, Xin Li^3^

Corresponding author：Xiaoqin Wang,Department of Gynecology, Affiliated Women and Children's Hospital of Ningbo University, 339 Liuting Street, Haishu District, Ningbo,315000, Zhejiang Province, China,

# ORCiD: https://orcid.org/0000-0002-5559-3646

# Tel:+8615968079043;E-mail:wxq021981@163.com

# **Supplementary Figure**


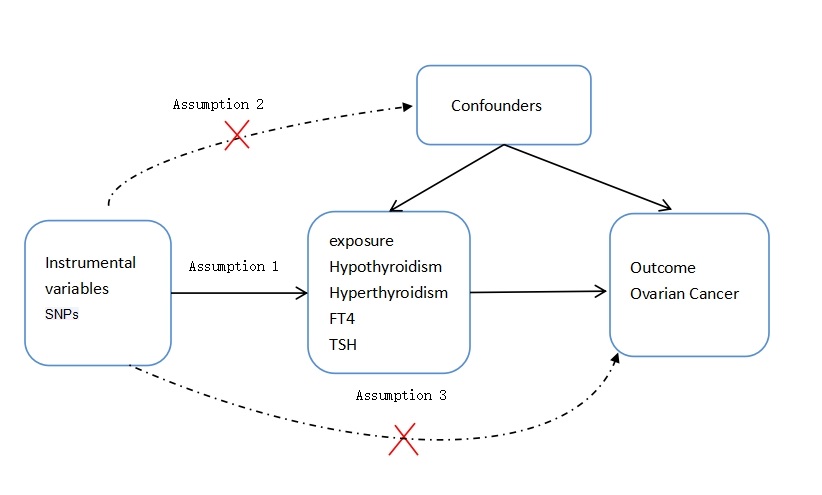


**Supplementary Figure 1**  Assumption 1: a robust correlation between instrumental variables and exposure, with SNPs being independent of each other. Assumption 2: Instrumental variables should not be associated with any factors that may confound the relationship between the exposure and outcome (ovarian cancer). Assumption 3: The influence of instrumental variables on outcomes should only occur through their effect on exposure, without any involvement of other causal pathway.


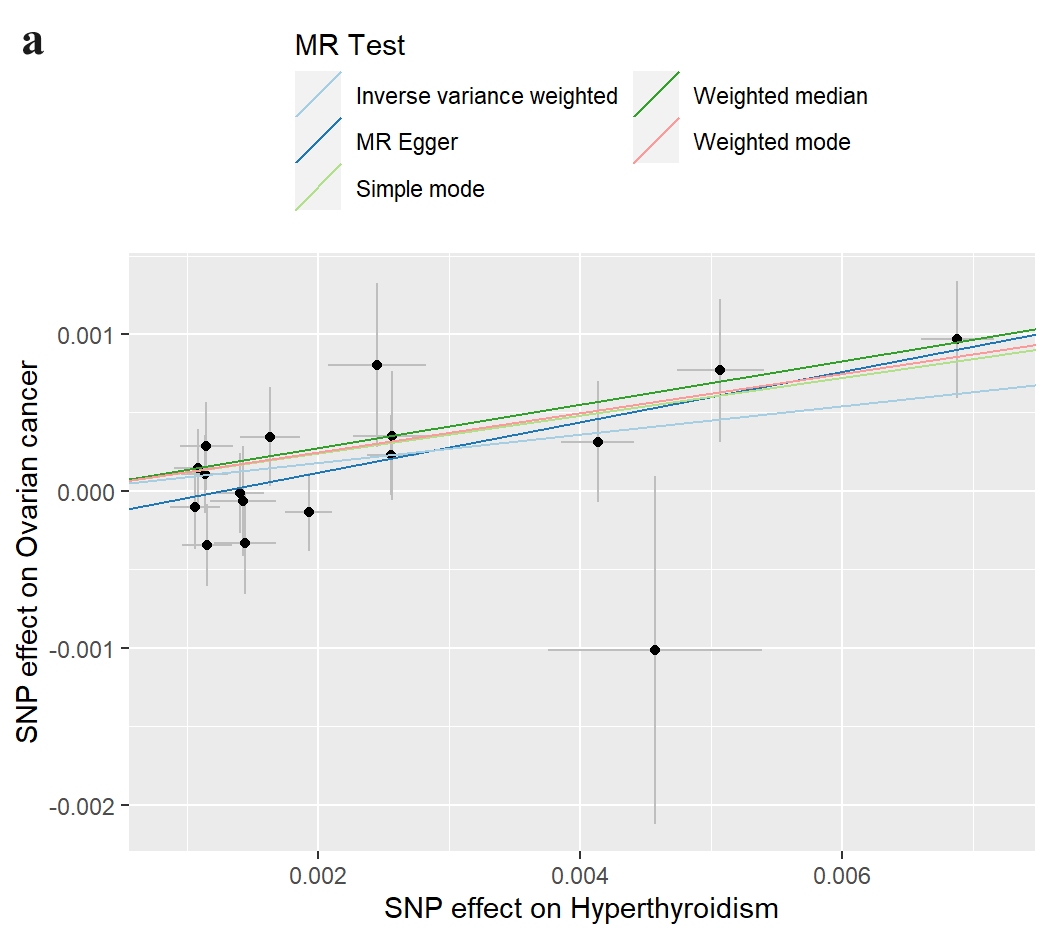
**
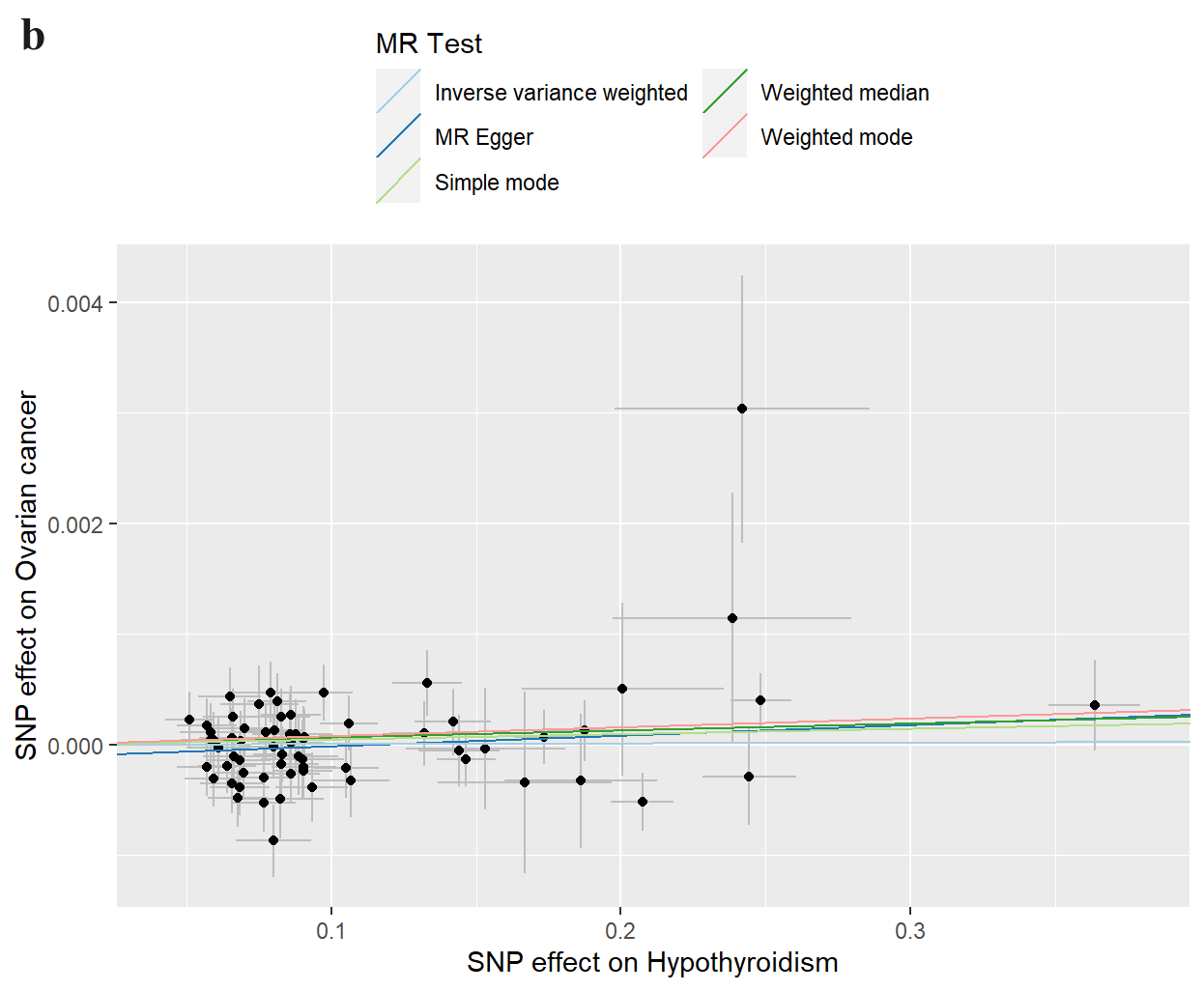
**

**
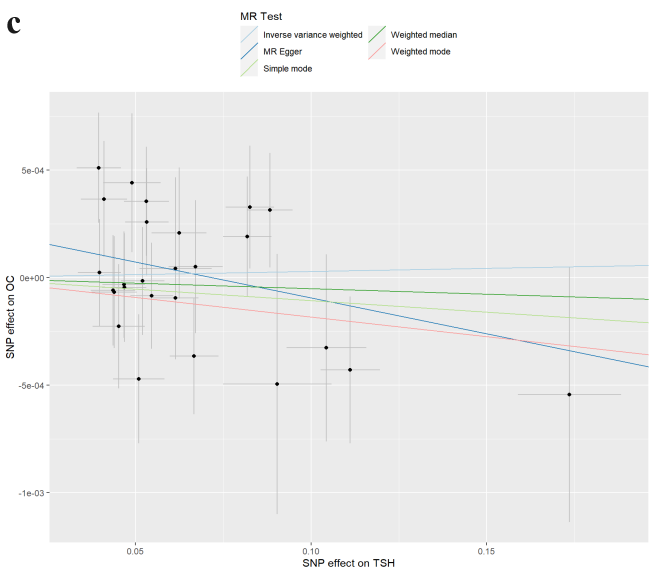

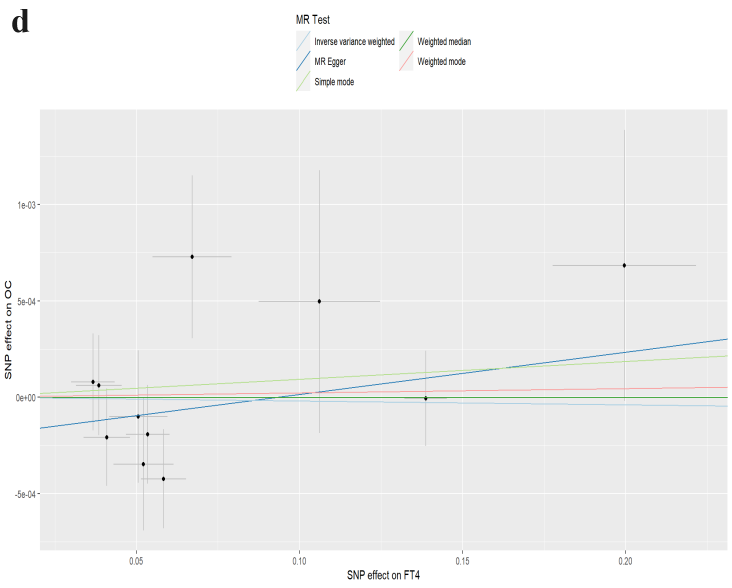
**

**Supplementary Figure 2** Scatter plots were used to analyze the causality of exposure (thyroid dysfunction)-outcome (ovarian cancer) associations through Mendelian randomization (MR) analyses. (**a**)Hyperthyroidism-OC.(**b**)Hypothyroidism-OC.(**c**)TSH-OC.(**d**)FT4-OC.


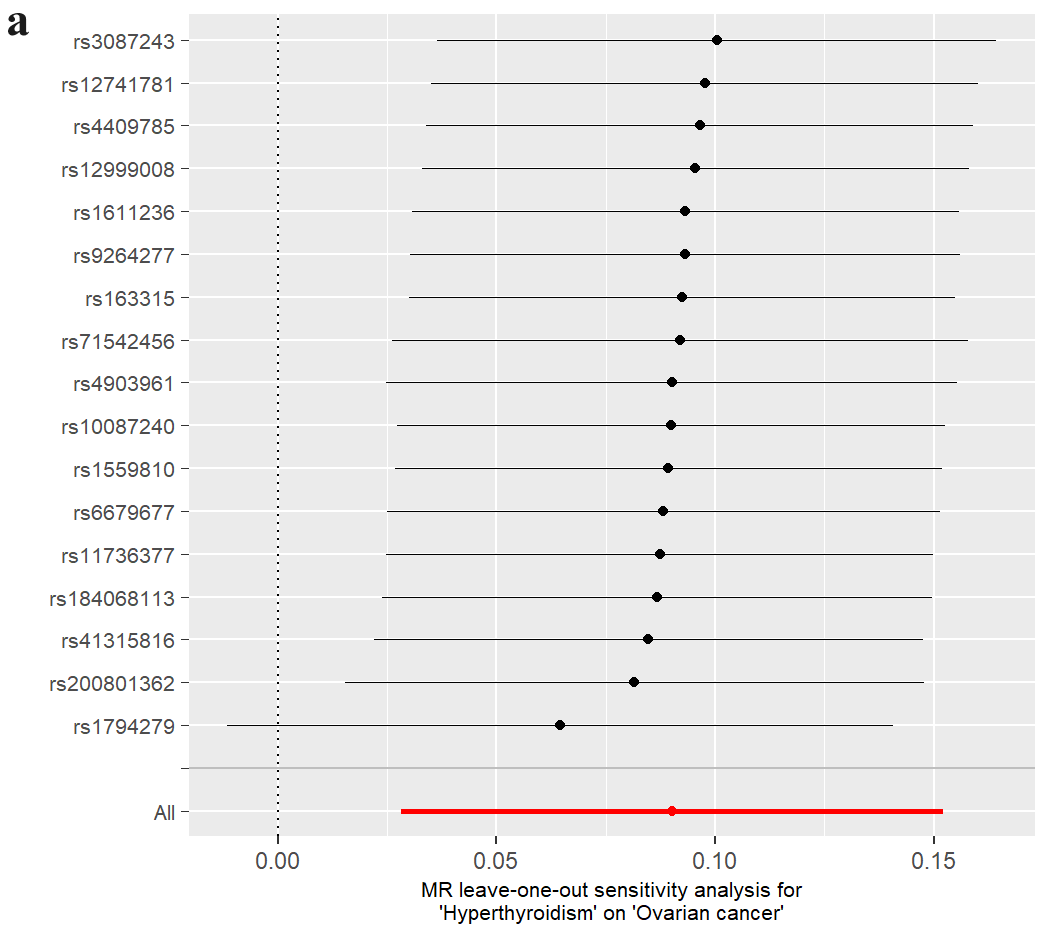

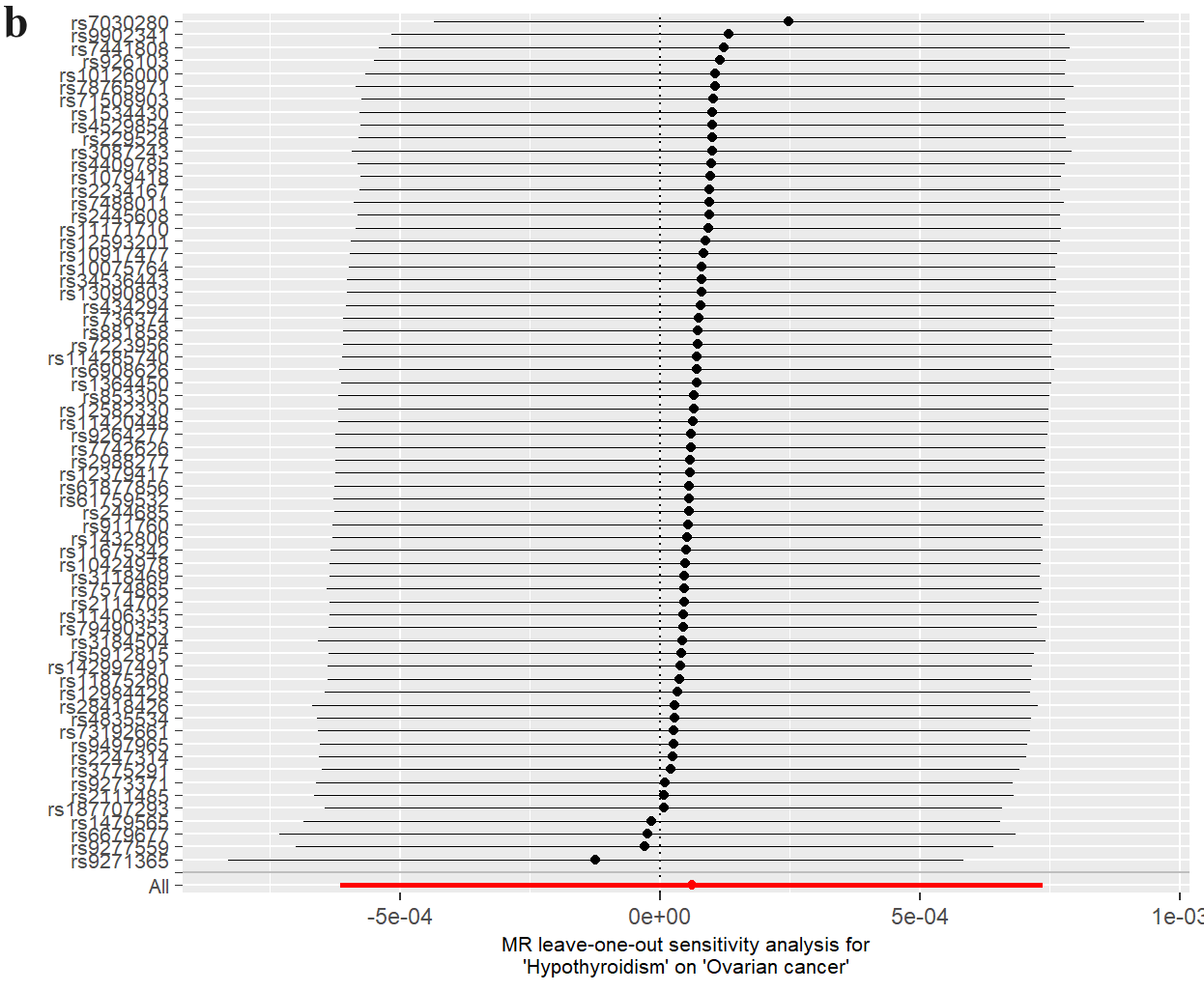


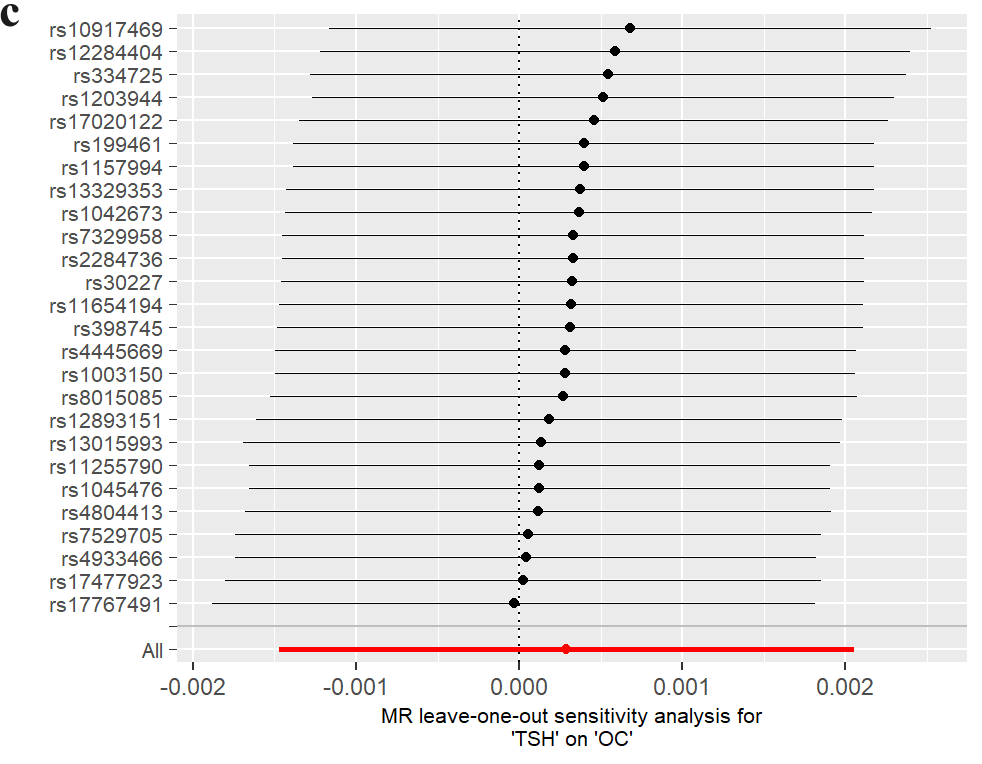

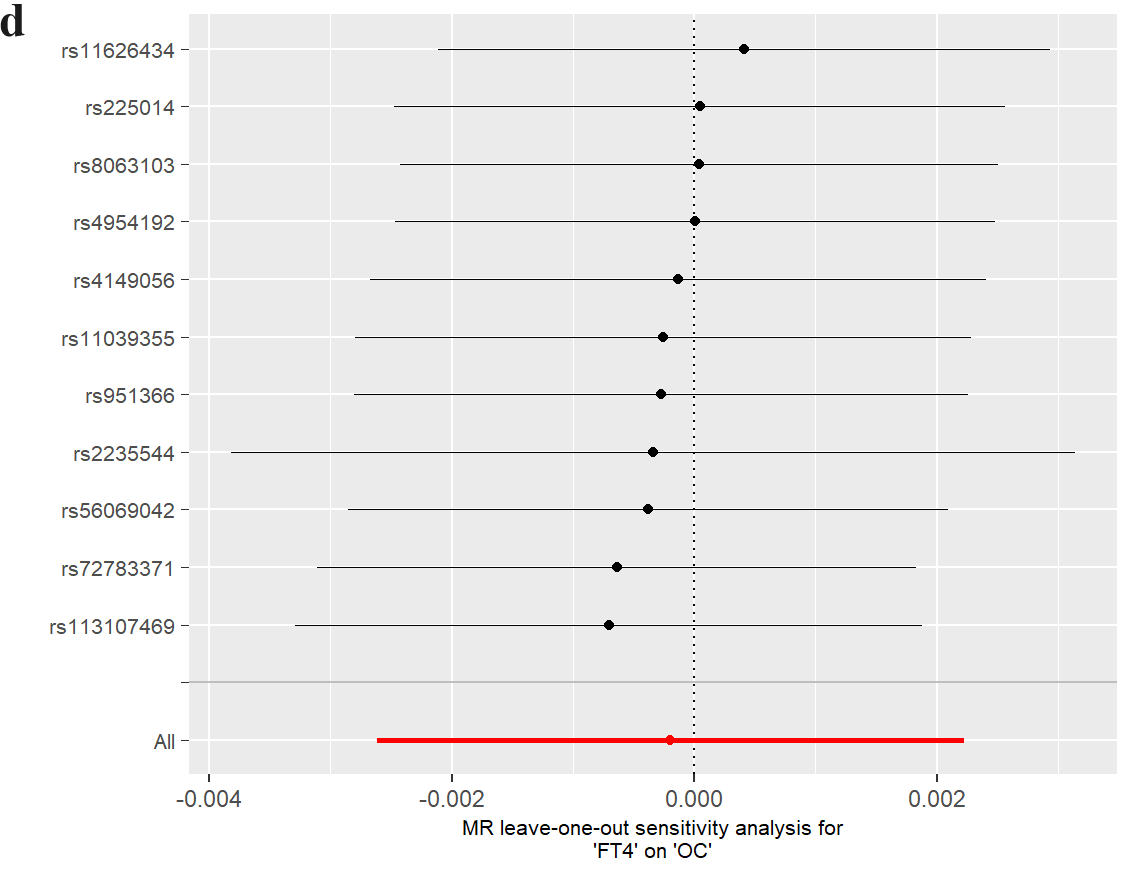


**Supplementary [Figure](https://www.ncbi.nlm.nih.gov/pmc/articles/PMC10030973/figure/f3/)3** The MR leave-one-out sensitivity analyses gradually eliminate a single SNP, and the remaining SNP results are shown in **(**a**)**Hyperthyroidism-OC.**(**b**)** Hypothyroidism-OC. **(**c**)** TSH-OC. **(**d**)** FT4-OC.


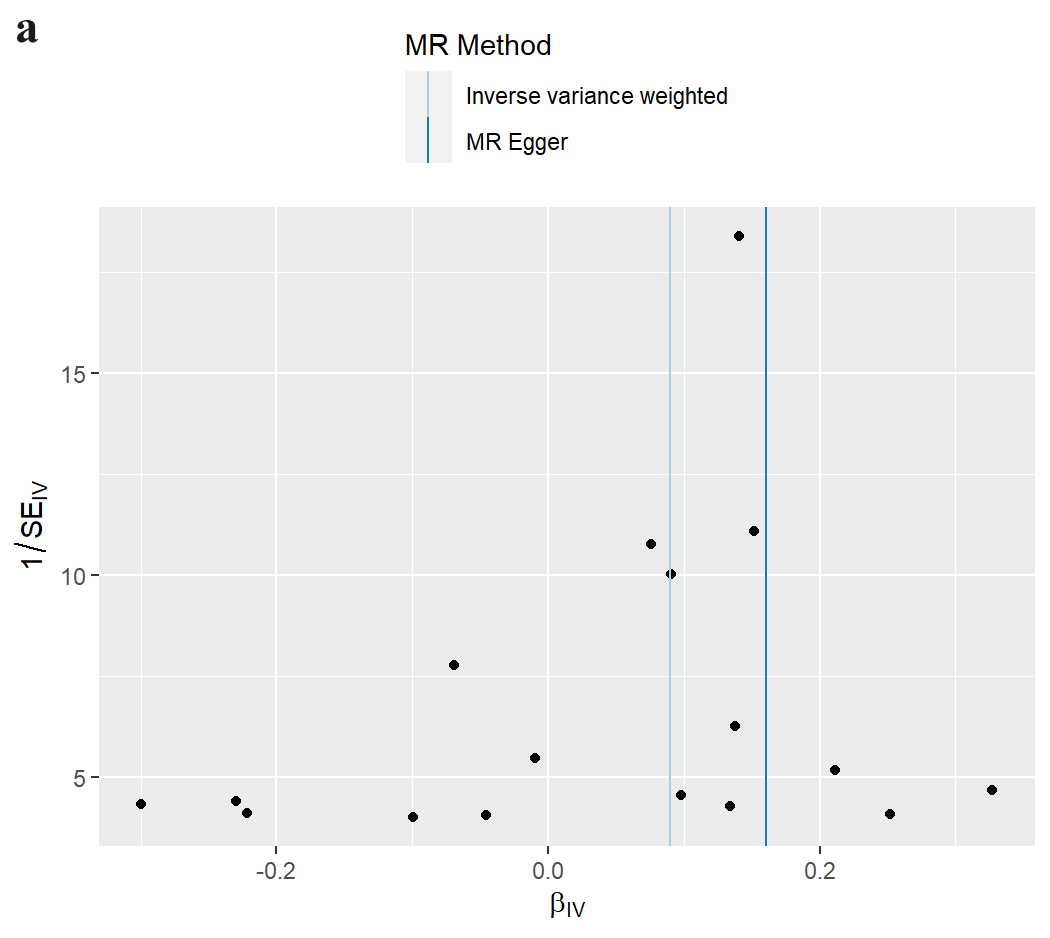

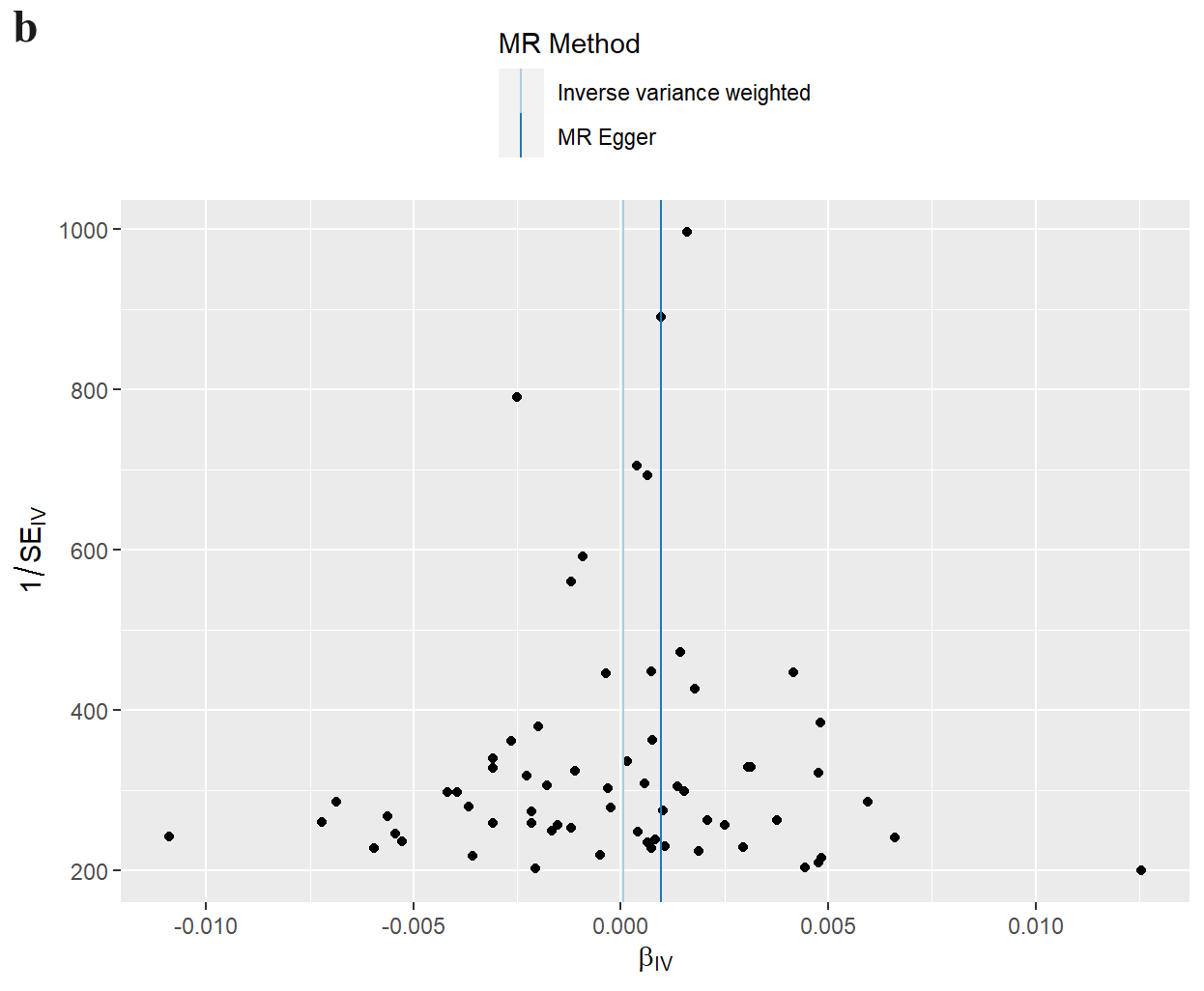


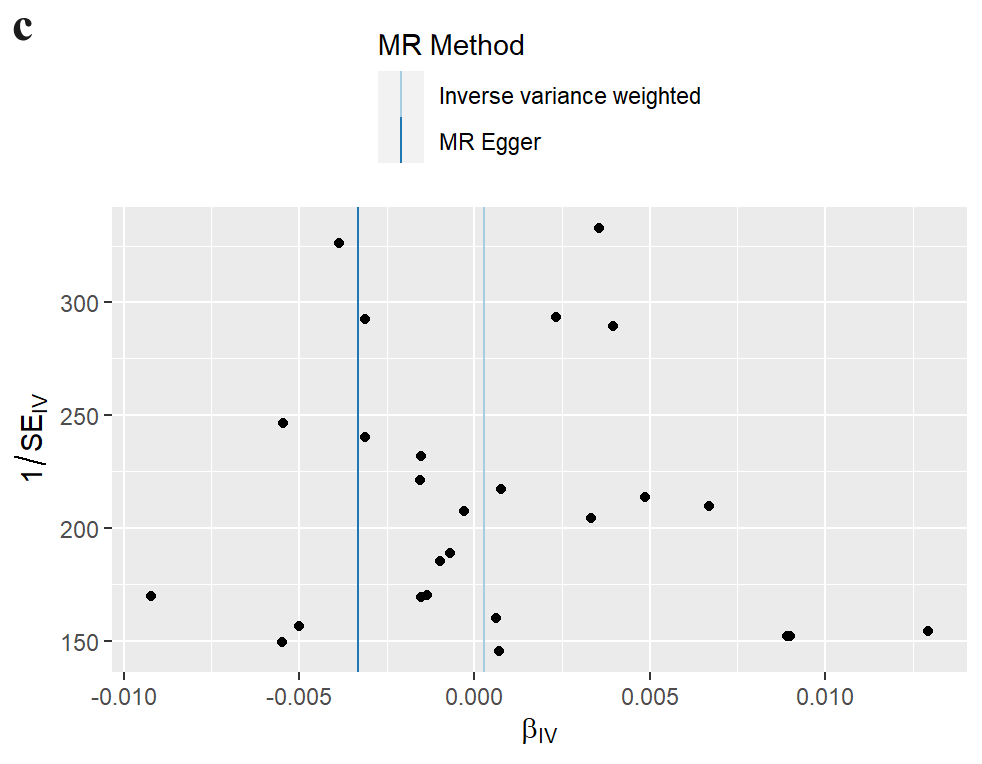

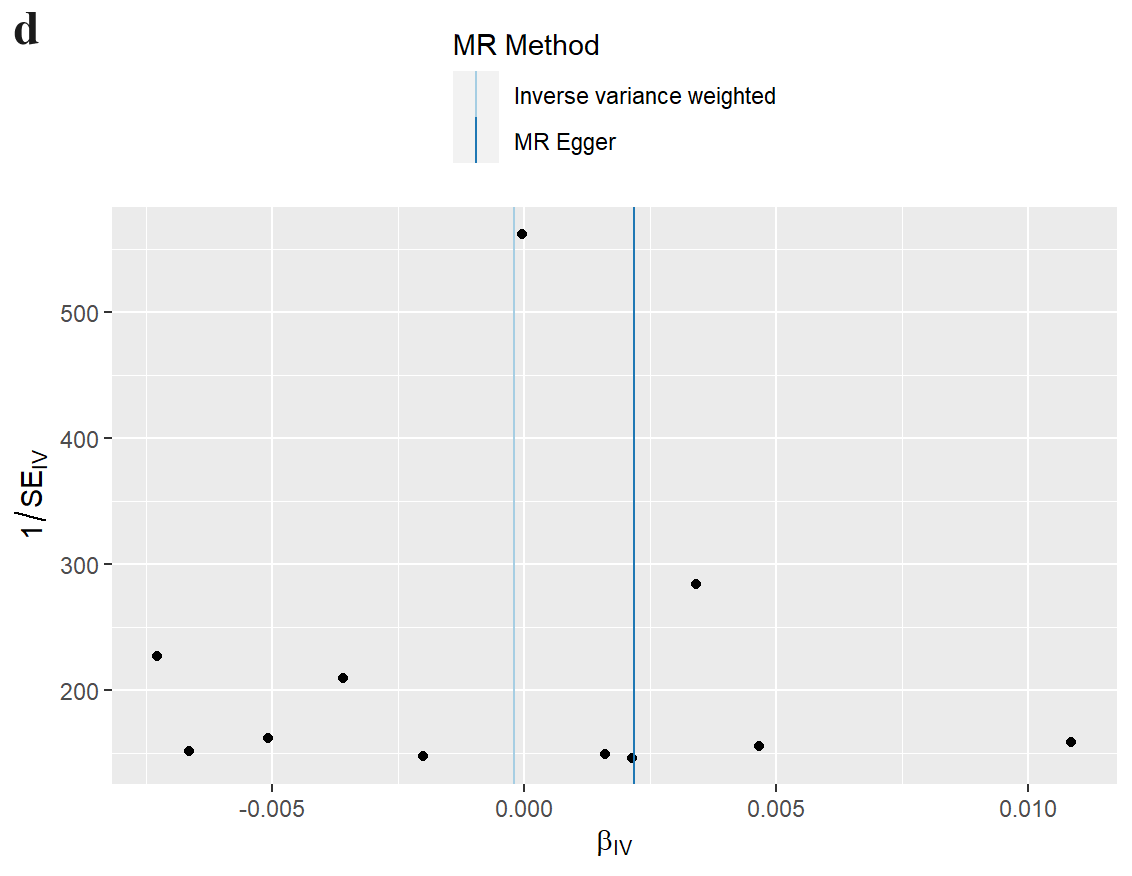


**Supplementary Figure 4** Funnel plots for Mendelian randomization (MR) analyses of the Causal relationship between thyroid dysfunction and Ovarian cancer.(**a**)Hyperthyroidism-OC.(**b**) Hypothyroidism-OC.(**c**)TSH-OC.(**d**)FT4-OC.


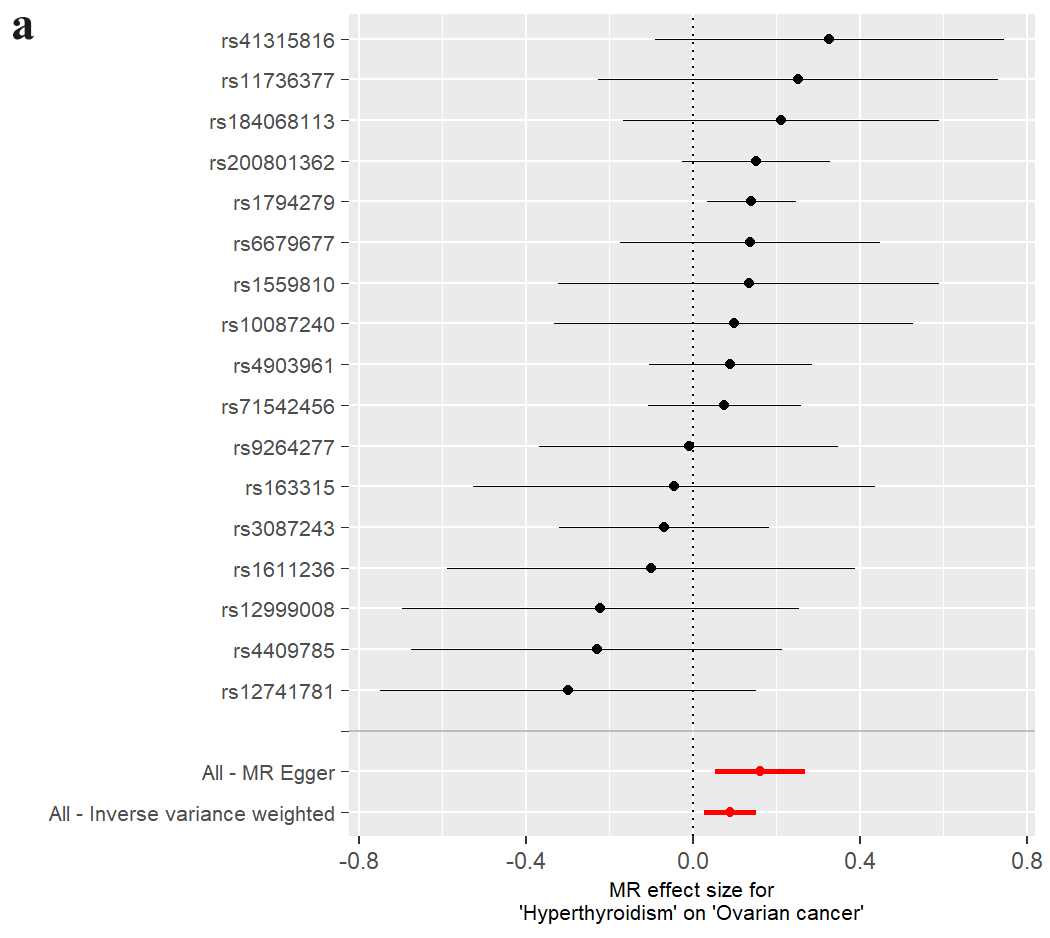

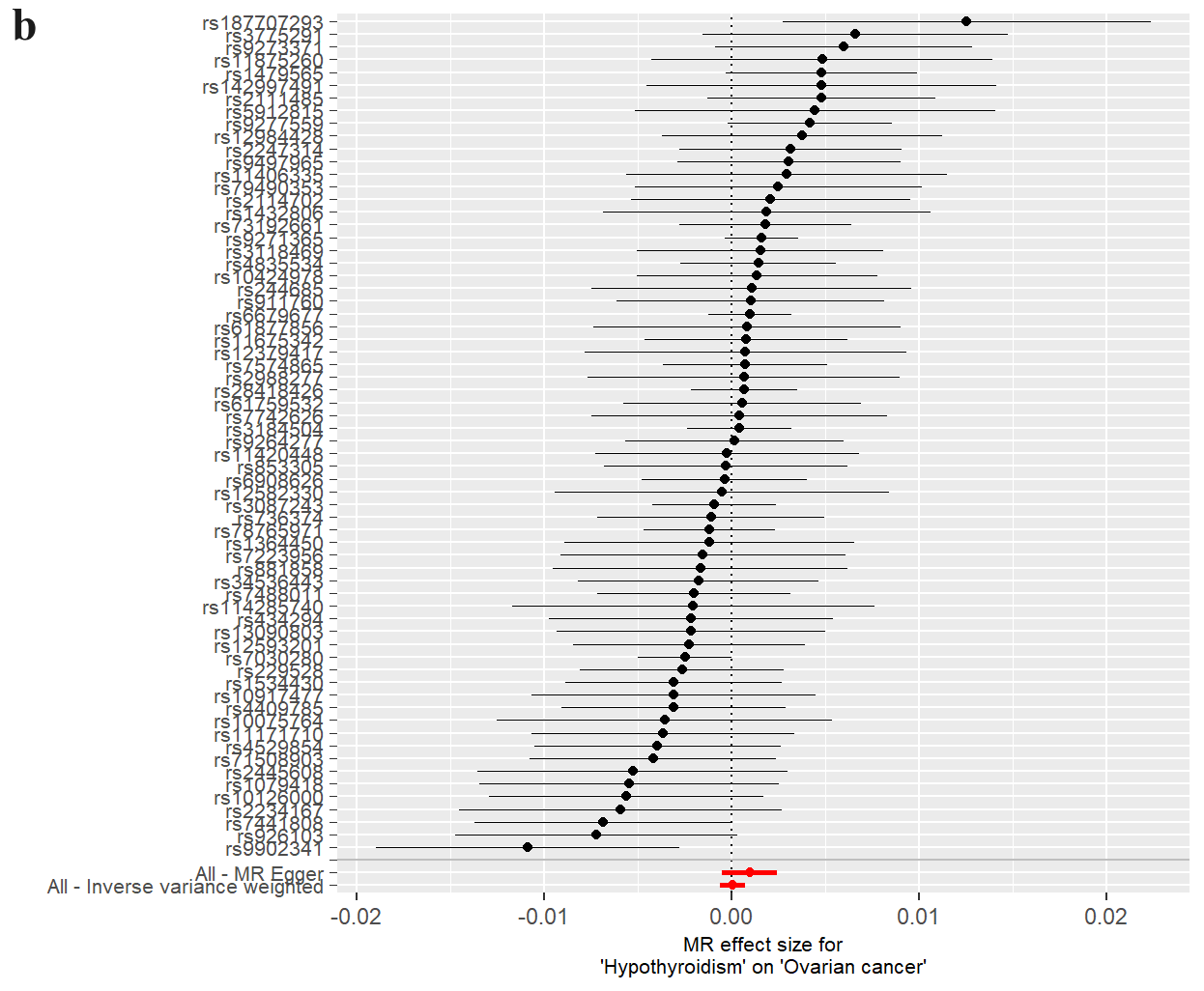


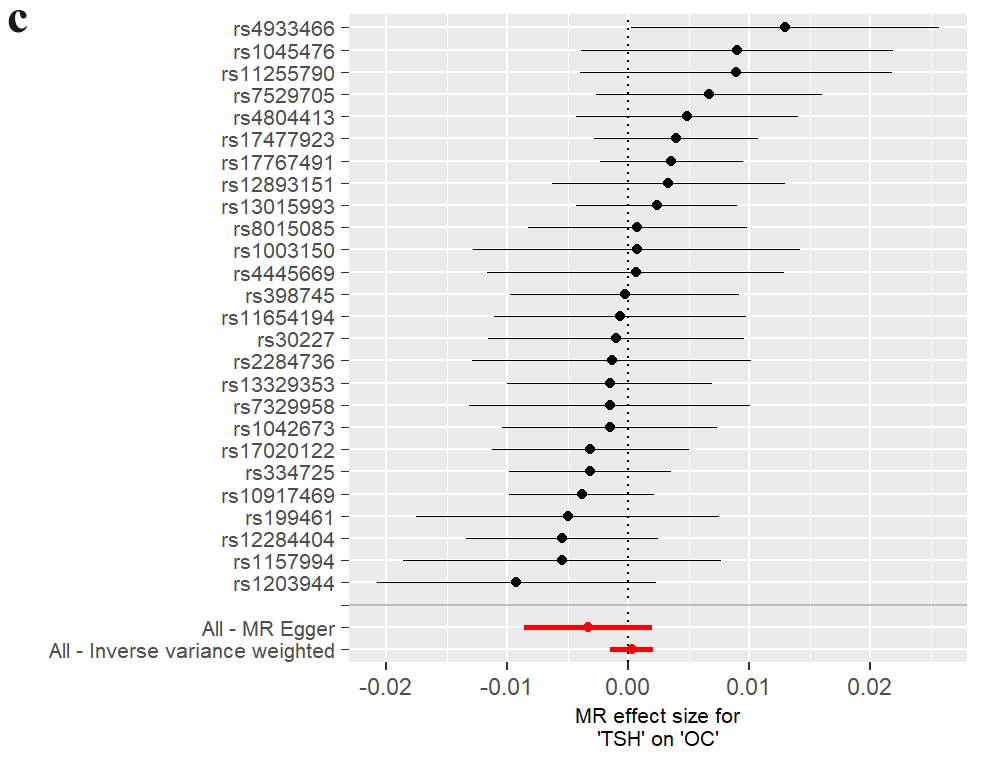

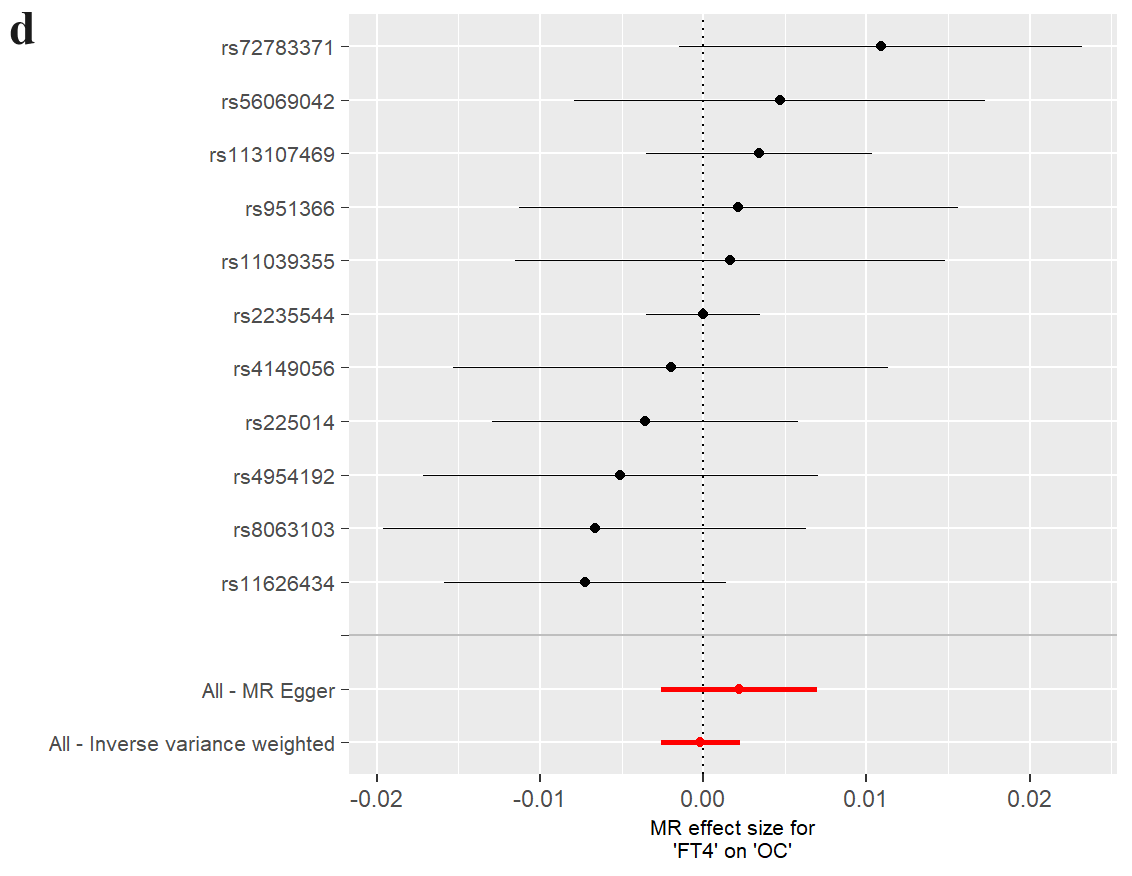


**Supplementary Figure 5** Forest plots for Mendelian randomization (MR) analyses of the Causal relationship between thyroid dysfunction and Ovarian cancer.(**a**)Hyperthyroidism-OC.(**b**) Hypothyroidism-OC. (**c**)TSH-OC.(**d**)FT4-OC
