# Supplementary material for: Causal relationship between thyroid dysfunction and ovarian cancer: a two-sample Mendelian randomization study
Source: BMC Cancer. 2024 May 23;24:629. doi: 10.1186/s12885-024-12385-5 (PMC11112802; doi:10.1186/s12885-024-12385-5)
Supplement: Supplementary file 3 — Supplementary Material 3 [file 12885_2024_12385_MOESM3_ESM.docx]

Supplementary Tables

# Article title:Causal relationship between thyroid dysfunction and ovarian cancer: A two-sample Mendelian randomization study

# Journal name: JOURNAL OF CANCER RESEARCH AND CLINICAL ONCOLOGY

Author names: Tingting Wang^1^, Xiaoqin Wang^2*^ ,Jun Wu^3^, Xin Li^3^

Corresponding author：Xiaoqin Wang,Department of Gynecology, Affiliated Women and Children's Hospital of Ningbo University, 339 Liuting Street, Haishu District, Ningbo,315000, Zhejiang Province, China,

# ORCiD: https://orcid.org/0000-0002-5559-3646

# Tel:+8615968079043;E-mail:wxq021981@163.com

# **Supplementary Tables**

**Supplementary Table S1 The characteristics of the selected SNPs as instrumental variable for Hypothyroidism and Ovarian cancer**

| Exposure | SNP^a^ | CHR^b^ | EA^c^ | OA^d^ | EAF^e^ | SE^f^ | β^h^ | *P*-val | F^g^ |
| --- | --- | --- | --- | --- | --- | --- | --- | --- | --- |
| Hypothyroidism | rs10075764 | 5 | G | A | 0.302413 | 0.0104 | -0.057 | 4.26E-08 | 562 |
|  | rs10126000 | 9 | A | C | 0.688692 | 0.0104 | -0.0683 | 5.13E-11 | 822 |
|  | rs10424978 | 19 | A | C | 0.624328 | 0.0102 | -0.0775 | 2.77E-14 | 1158 |
|  | rs1079418 | 6 | G | A | 0.262374 | 0.011 | -0.0657 | 2.14E-09 | 686 |
|  | rs10917477 | 1 | G | A | 0.386901 | 0.01 | 0.064 | 1.75E-10 | 798 |
|  | rs11171710 | 12 | A | G | 0.443916 | 0.01 | -0.0698 | 3.19E-12 | 988 |
|  | rs11406335 | 13 | G | T | 0.417169 | 0.0103 | -0.057 | 3.44E-08 | 649 |
|  | rs11420448 | 2 | T | G | 0.0467006 | 0.0275 | 0.1533 | 2.39E-08 | 860 |
|  | rs114285740 | 1 | C | G | 0.0273776 | 0.0301 | 0.1669 | 3.06E-08 | 609 |
|  | rs11675342 | 2 | T | C | 0.387729 | 0.01 | 0.0906 | 1.40E-19 | 1604 |
|  | rs11875260 | 18 | G | A | 0.172925 | 0.0135 | 0.0751 | 2.54E-08 | 662 |
|  | rs12379417 | 9 | A | G | 0.317559 | 0.0103 | 0.0583 | 1.51E-08 | 605 |
|  | rs12582330 | 12 | T | G | 0.624365 | 0.0109 | -0.061 | 2.05E-08 | 717 |
|  | rs12593201 | 15 | A | G | 0.325432 | 0.0112 | 0.0905 | 7.69E-16 | 1480 |
|  | rs12984428 | 19 | A | G | 0.35604 | 0.0102 | -0.0659 | 1.11E-10 | 818 |
|  | rs13090803 | 3 | T | G | 0.190624 | 0.0128 | 0.0829 | 9.00E-11 | 871 |
|  | rs1364450 | 6 | C | A | 0.126235 | 0.0139 | 0.0886 | 1.97E-10 | 711 |
|  | rs142997491 | 16 | G | A | 0.0135637 | 0.0412 | 0.2385 | 7.02E-09 | 625 |
|  | rs1432806 | 5 | G | A | 0.338619 | 0.0105 | 0.0583 | 2.89E-08 | 625 |
|  | rs1479565 | 5 | A | G | 0.498866 | 0.0101 | 0.0975 | 7.53E-22 | 1958 |
|  | rs1534430 | 2 | T | C | 0.428394 | 0.0101 | -0.086 | 1.44E-17 | 1490 |
|  | rs187707293 | 4 | A | T | 0.0133864 | 0.044 | 0.2419 | 3.99E-08 | 634 |
|  | rs2111485 | 2 | G | A | 0.479023 | 0.0102 | 0.0813 | 1.43E-15 | 1357 |
|  | rs2114702 | 14 | A | T | 0.26671 | 0.0111 | 0.07 | 3.00E-10 | 787 |
|  | rs2234167 | 1 | A | G | 0.102013 | 0.015 | 0.0825 | 3.75E-08 | 512 |
|  | rs2247314 | 6 | C | T | 0.376495 | 0.0104 | -0.086 | 1.06E-16 | 1429 |
|  | rs229528 | 22 | T | C | 0.496016 | 0.01 | 0.0903 | 2.31E-19 | 1678 |
|  | rs2445608 | 8 | A | G | 0.430366 | 0.0101 | -0.0593 | 3.79E-09 | 708 |
|  | rs244685 | 5 | G | T | 0.79915 | 0.0132 | -0.0858 | 7.06E-11 | 971 |
|  | rs28418426 | 6 | C | T | 0.501169 | 0.0133 | 0.1877 | 2.21E-45 | 7354 |
|  | rs2988277 | 1 | T | C | 0.287022 | 0.0106 | 0.0593 | 2.49E-08 | 591 |
|  | rs3087243 | 2 | A | G | 0.386088 | 0.0102 | -0.1466 | 4.77E-47 | 4221 |
|  | rs3118469 | 10 | T | A | 0.295174 | 0.0106 | 0.0803 | 3.82E-14 | 1103 |
|  | rs3184504 | 12 | C | T | 0.668513 | 0.0102 | -0.1734 | 7.50E-65 | 5539 |
|  | rs34536443 | 19 | C | G | 0.0440279 | 0.0263 | -0.1863 | 1.46E-12 | 1201 |
|  | rs3775291 | 4 | T | C | 0.287788 | 0.0108 | -0.0649 | 1.65E-09 | 709 |
|  | rs434294 | 5 | G | A | 0.322802 | 0.0109 | -0.0683 | 3.36E-10 | 838 |
|  | rs4409785 | 11 | C | T | 0.142566 | 0.0133 | 0.1069 | 8.04E-16 | 1149 |
|  | rs4529854 | 10 | T | C | 0.723438 | 0.0107 | -0.0768 | 6.56E-13 | 970 |
|  | rs4835534 | 4 | C | T | 0.156356 | 0.0132 | -0.1421 | 7.06E-27 | 2196 |
|  | rs5912815 | X | G | T | 0.576761 | 0.0084 | -0.0511 | 1.05E-09 | 523 |
|  | rs61759532 | 17 | T | C | 0.188941 | 0.0122 | 0.0905 | 1.42E-13 | 1032 |
|  | rs61877856 | 11 | T | C | 0.197495 | 0.0115 | -0.0658 | 1.14E-08 | 563 |
|  | rs6679677 | 1 | A | C | 0.108444 | 0.0159 | 0.3637 | 2.39E-115 | 10766 |
|  | rs6908626 | 6 | T | G | 0.17075 | 0.0141 | 0.1441 | 2.04E-24 | 2426 |
|  | rs7030280 | 9 | T | C | 0.745664 | 0.0108 | 0.2075 | 1.02E-82 | 6809 |
|  | rs71508903 | 10 | T | C | 0.210816 | 0.0125 | 0.0934 | 9.34E-14 | 1193 |
|  | rs7223956 | 17 | C | T | 0.897005 | 0.0144 | -0.0902 | 4.27E-10 | 617 |
|  | rs73192661 | 3 | T | C | 0.428858 | 0.01 | -0.1061 | 4.05E-26 | 2274 |
|  | rs736374 | 11 | A | G | 0.375103 | 0.0103 | 0.0832 | 6.00E-16 | 1335 |
|  | rs7441808 | 4 | G | A | 0.21091 | 0.0111 | 0.0766 | 5.17E-12 | 802 |
|  | rs7488011 | 12 | T | C | 0.356485 | 0.0111 | 0.1052 | 2.52E-21 | 2093 |
|  | rs7574865 | 2 | G | T | 0.74252 | 0.0117 | -0.1321 | 1.67E-29 | 2755 |
|  | rs7742626 | 6 | C | T | 0.267343 | 0.0116 | 0.0686 | 3.41E-09 | 757 |
|  | rs78765971 | 1 | G | A | 0.146942 | 0.0162 | 0.2444 | 1.68E-51 | 6235 |
|  | rs79490353 | 13 | C | T | 0.0241151 | 0.0349 | 0.2006 | 8.82E-09 | 778 |
|  | rs853305 | 8 | C | T | 0.718894 | 0.0111 | -0.0802 | 4.40E-13 | 1068 |
|  | rs881858 | 6 | A | G | 0.747899 | 0.0108 | 0.0665 | 8.46E-10 | 685 |
|  | rs911760 | 9 | A | C | 0.212143 | 0.0125 | 0.0879 | 1.95E-12 | 1062 |
|  | rs926103 | 1 | C | T | 0.697985 | 0.0104 | -0.0678 | 7.65E-11 | 796 |
|  | rs9264277 | 6 | C | T | 0.645071 | 0.0111 | -0.0862 | 9.03E-15 | 1400 |
|  | rs9271365 | 6 | G | T | 0.443466 | 0.0105 | 0.2484 | 4.91E-123 | 12883 |
|  | rs9273371 | 6 | T | C | 0.193385 | 0.014 | 0.0791 | 1.65E-08 | 802 |
|  | rs9277559 | 6 | C | T | 0.331026 | 0.012 | -0.133 | 1.86E-28 | 3238 |
|  | rs9497965 | 6 | T | C | 0.409035 | 0.0102 | 0.0827 | 3.71E-16 | 1360 |
|  | rs9902341 | 17 | T | C | 0.172257 | 0.0129 | 0.0801 | 4.68E-10 | 751 |

a: single nucleotide polymorphism;b:chromosome; c: effect allele exposure; d: other allele exposure; e:effect allele frequency,f: standard error;g:F value;h:estimate of the causal effect.

**Supplementary Table S2 The characteristics of the selected SNPs as instrumental variable for Hyperthyroidism and Ovarian cancer**

| Exposure | SNP^a^ | CHR^b^ | EA^c^ | OA^d^ | EAF^e^ | SE^f^ | β^h^ | *P*-val | F^g^ |
| --- | --- | --- | --- | --- | --- | --- | --- | --- | --- |
| Hyperthyroidism | rs10087240 | 8 | T | C | 0.458311 | 0.00018 | 0.001134 | 2.30E-10 | 39 |
|  | rs11736377 | 4 | T | C | 0.737388 | 0.000203 | -0.00115 | 1.40E-08 | 31 |
|  | rs12741781 | 1 | G | T | 0.328488 | 0.000191 | 0.001148 | 1.90E-09 | 36 |
|  | rs12999008 | 2 | A | T | 0.01272 | 0.000818 | 0.004573 | 2.70E-08 | 31 |
|  | rs1559810 | 3 | A | C | 0.406014 | 0.000182 | 0.00108 | 2.20E-09 | 35 |
|  | rs1611236 | 6 | A | G | 0.674823 | 0.000191 | -0.00106 | 1.80E-09 | 30 |
|  | rs163315 | 5 | T | G | 0.145897 | 0.000254 | 0.001425 | 2.30E-08 | 31 |
|  | rs1794279 | 6 | T | G | 0.120389 | 0.000277 | 0.006884 | 6.90E-134 | 619 |
|  | rs184068113 | 6 | C | T | 0.401785 | 0.000229 | 0.001635 | 1.70E-13 | 50 |
|  | rs200801362 | 6 | C | T | 0.157012 | 0.000333 | 0.005073 | 1.60E-50 | 232 |
|  | rs3087243 | 2 | A | G | 0.448193 | 0.00018 | -0.00193 | 9.00E-27 | 114 |
|  | rs41315816 | 6 | C | T | 0.061221 | 0.000372 | -0.00245 | 2.00E-08 | 43 |
|  | rs4409785 | 11 | C | T | 0.171039 | 0.000237 | 0.00144 | 1.10E-09 | 36 |
|  | rs4903961 | 14 | G | C | 0.376258 | 0.000185 | 0.002559 | 1.40E-43 | 191 |
|  | rs6679677 | 1 | A | C | 0.096716 | 0.000302 | 0.002565 | 1.60E-17 | 72 |
|  | rs71542456 | 6 | G | A | 0.200752 | 0.00028 | 0.004136 | 1.10E-47 | 218 |
|  | rs9264277 | 6 | C | T | 0.634299 | 0.000185 | 0.001402 | 3.00E-17 | 57 |

a: single nucleotide polymorphism;b:chromosome; c: effect allele exposure; d: other allele exposure; e:effect allele frequency,f: standard error;g:F value;h:estimate of the causal effect.

**Supplementary Table S3 The characteristics of the selected SNPs as instrumental variable for FT4 and Ovarian cancer**

| Exposure | SNP^a^ | CHR^b^ | EA^c^ | OA^d^ | EAF^e^ | SE^f^ | β^h^ | *P*-val | F^g^ |
| --- | --- | --- | --- | --- | --- | --- | --- | --- | --- |
| FT4 | rs11039355 | 11 | T | C | 0.283 | 0.007 | -0.0385 | 3.51E-08 | 43 |
|  | rs113107469 | 18 | T | C | 0.01465 | 0.022 | 0.1996 | 1.00E-19 | 83 |
|  | rs11626434 | 14 | C | G | 0.3741 | 0.0069 | 0.0583 | 4.08E-17 | 115 |
|  | rs2235544 | 1 | A | C | 0.4432 | 0.0065 | 0.1387 | 4.20E-10 | 691 |
|  | rs225014 | 14 | T | C | 0.5788 | 0.0067 | 0.0535 | 1.83E-15 | 100 |
|  | rs4149056 | 12 | T | C | 0.8773 | 0.0089 | -0.0506 | 1.34E-08 | 39 |
|  | rs4954192 | 2 | T | C | 0.6593 | 0.0071 | -0.0409 | 8.38E-09 | 54 |
|  | rs56069042 | 18 | A | G | 0.98031 | 0.0186 | 0.1061 | 1.16E-08 | 31 |
|  | rs72783371 | 10 | A | C | 0.8974 | 0.0121 | 0.0671 | 2.77E-08 | 59 |
|  | rs8063103 | 16 | C | G | 0.8457 | 0.0092 | -0.0522 | 1.61E-08 | 51 |
|  | rs951366 | 1 | T | C | 0.647 | 0.0067 | 0.0367 | 4.39E-08 | 44 |

a: single nucleotide polymorphism;b:chromosome; c: effect allele exposure; d: other allele exposure; e:effect allele frequency,f: standard error;g:F value;h:estimate of the causal effect.

**Supplementary Table S4 The characteristics of the selected SNPs as instrumental variable for TSH and Ovarian cancer**

| Exposure | SNP^a^ | CHR^b^ | EA^c^ | OA^d^ | EAF^e^ | SE^f^ | β^h^ | pval | F^g^ |
| --- | --- | --- | --- | --- | --- | --- | --- | --- | --- |
| TSH | rs1003150 | 14 | A | T | 0.8777 | 0.0103 | -0.0614 | 2.87E-09 | 58 |
|  | rs1042673 | 17 | A | G | 0.4098 | 0.0061 | -0.0546 | 3.57E-19 | 104 |
|  | rs1045476 | 16 | A | G | 0.3686 | 0.0082 | 0.049 | 2.36E-09 | 80 |
|  | rs10917469 | 1 | A | G | 0.8269 | 0.0085 | 0.1112 | 3.95E-39 | 256 |
|  | rs11255790 | 10 | T | C | 0.1937 | 0.0066 | -0.041 | 6.83E-10 | 37 |
|  | rs1157994 | 17 | A | G | 0.03617 | 0.0155 | -0.0904 | 5.28E-09 | 41 |
|  | rs11654194 | 17 | A | G | 0.2756 | 0.0061 | 0.0467 | 1.82E-14 | 62 |
|  | rs1203944 | 20 | T | C | 0.2775 | 0.0073 | -0.0509 | 2.42E-12 | 75 |
|  | rs12284404 | 11 | A | G | 0.1722 | 0.0069 | -0.0667 | 2.48E-22 | 91 |
|  | rs12893151 | 14 | A | C | 0.1795 | 0.0078 | -0.0624 | 1.02E-15 | 82 |
|  | rs13015993 | 2 | A | G | 0.6016 | 0.0069 | 0.0818 | 4.52E-32 | 232 |
|  | rs13329353 | 15 | T | C | 0.658 | 0.0065 | 0.0614 | 5.17E-21 | 122 |
|  | rs17020122 | 1 | T | C | 0.1424 | 0.0114 | 0.1044 | 5.32E-20 | 192 |
|  | rs17477923 | 15 | T | C | 0.8274 | 0.0069 | 0.0826 | 2.57E-33 | 140 |
|  | rs17767491 | 16 | A | G | 0.7463 | 0.0065 | 0.0883 | 3.35E-42 | 213 |
|  | rs199461 | 17 | A | G | 0.4125 | 0.0074 | 0.0452 | 1.13E-09 | 71 |
|  | rs2284736 | 14 | A | G | 0.4528 | 0.0064 | 0.0436 | 1.06E-11 | 68 |
|  | rs30227 | 16 | T | C | 0.6245 | 0.0063 | -0.0468 | 7.59E-14 | 74 |
|  | rs334725 | 1 | A | G | 0.92399 | 0.0147 | 0.1737 | 2.45E-32 | 307 |
|  | rs398745 | 14 | A | C | 0.5192 | 0.0062 | -0.052 | 3.97E-17 | 97 |
|  | rs4445669 | 11 | T | C | 0.6213 | 0.0061 | -0.0397 | 5.76E-11 | 53 |
|  | rs4804413 | 19 | T | C | 0.3654 | 0.0062 | 0.0532 | 8.64E-18 | 94 |
|  | rs4933466 | 10 | A | G | 0.5513 | 0.0063 | 0.0395 | 5.13E-10 | 55 |
|  | rs7329958 | 13 | T | C | 0.2683 | 0.0065 | -0.0439 | 1.13E-11 | 54 |
|  | rs7529705 | 1 | A | G | 0.3439 | 0.0064 | 0.0531 | 1.39E-16 | 91 |
|  | rs8015085 | 14 | A | G | 0.2097 | 0.0077 | 0.0671 | 2.45E-18 | 107 |

a: single nucleotide polymorphism;b:chromosome; c: effect allele exposure; d: other allele exposure; e:effect allele frequency,f: standard error;g:F value;h:estimate of the causal effect.
